# Gender differences in suicide and suicide attempts among patients in AUD treatment

**DOI:** 10.1192/j.eurpsy.2023.163

**Published:** 2023-07-19

**Authors:** J. G. Bramness

**Affiliations:** Department of alcohol, drug and tobacco research, Norwegian Institute of Public Helath, Oslo, Norway

## Abstract

**Abstract:**

Alcohol plays a part in suicide risk in two ways. Alcohol intoxication is often a preceding factor in the acute suicidal phase. But also, chronic overuse or Alcohol Use Disorder (AUD) is a risk factor for suicide attempts and suicides over time. At least 1 in 4 AUD patients have serious lifetime suicide attempts. Standardized mortality rates (SMR) for suicide in AUD lie around 15-25. Gender differences in suicide risk and suicide attempts are influenced by at least 2 phenomena. Firstly, suicide attempts are more common and competed suicide is less common among females in the general population. Secondly, female AUD is rarer and female AUD patients tend to be sicker and have more mental health co-morbidities than male AUD patients. This leads to SMRs for suicide in female AUD patients being higher than in male AUD patients, even if suicides are more common in male AUD patients. Also, for suicide attempts, these are more common in female AUD patients. Suicide attempts seem to be more related to AUD severity in male AUD patients, but more related to mental heath co-morbidities in female AUD patients.

**Disclosure of Interest:**

None Declared

